# Precision gene editing technology to modulate genes in oral diseases: Therapeutic applications and delivery strategies

**DOI:** 10.1016/j.jgeb.2026.100830

**Published:** 2026-09-28

**Authors:** Yihan Wang, Xuesong Li, Chunpeng Liu, Bing He, Yiming Liu, Qingyan Liu, Chenyu Liu, Daiyun Chen, Shufang Wang, Lin Zhang

**Affiliations:** a School of Stomatology, Shandong First Medical University & Shandong Academy of Medical Sciences, Jinan, Shandong 250021, China; b Department of Stomatology, The 960th Hospital of the PLA Joint Logistics Support Force, Jinan, Shandong 250031, China; c College of Clinical and Basic Medicine (Institute of Basic Medicine), Shandong First Medical University & Shandong Academy of Medical Sciences, Jinan, Shandong 250117, China; d Key Laboratory of Bioactive Materials for Ministry of Education, College of Life Sciences, Nankai University, Tianjin 300071, China

**Keywords:** Oral disease treatments, Gene editing technology, CRISPR-Cas9, Delivery systems

## Abstract

Oral diseases represent a significant global health burden. Given the limited therapeutic efficacy of conventional mechanical interventions and antimicrobial treatments, there is a critical demand for targeted, precision therapies. Gene editing has emerged as a revolutionary approach that modifies specific genes to alter cellular phenotypes, leading to significant advances in human medicine. This comprehensive narrative review evaluates the current state and developmental trends of gene editing, focusing on its transformative potential in oral disease management. This outlines the fundamental molecular mechanisms of the gene editing system and comprehensively synthesizes its cutting-edge therapeutic applications across diverse oral pathologies, including dental caries, oral squamous cell carcinoma (OSCC), and maxillofacial bone defects. Furthermore, to overcome the unique anatomical barriers of the oral cavity, advanced biomedical delivery strategies—including physical methods, viral vectors, nanoparticle carriers, hydrogels, and fiber membranes—are critically evaluated. However, current evidence remains predominantly preclinical, and major translational barriers, especially safe delivery and off-target effects, must be overcome before clinical application. In conclusion, gene editing technology, particularly the CRISPR-Cas9 system, enables the targeted modulation of gene expression, offers deeper insights into oral disease progression, and facilitates the development of innovative therapeutic strategies.

## Highlights

• Comprehensive design principles of gene editing technology were introduced.
• Gene editing technology could treat various oral diseases.
• A variety of gene editing delivery methods were described.
• The application status of gene editing for oral disease treatments was clarified.

## Graphical abstract

**Figure:**
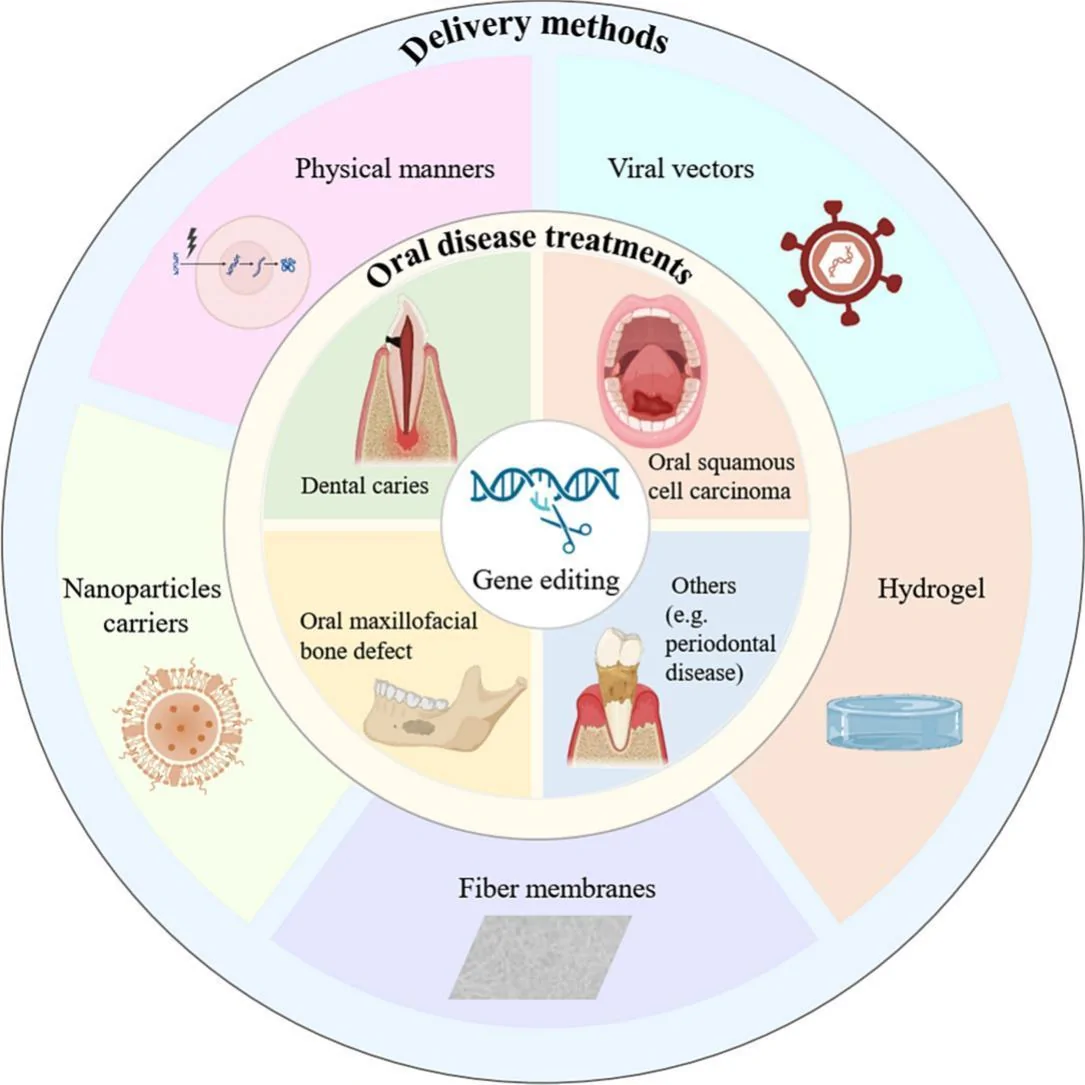

## 1 Introduction

Oral diseases are among the most prevalent global health conditions, affecting approximately 3.5 billion people worldwide.^1^ Severe oral diseases not only significantly impact overall health but are also associated with major systemic illnesses, including cardiovascular disease, diabetes, and arthritis.^2^ Particularly for prevalent conditions such as periodontal and peri-implant diseases, contemporary clinical management is anchored in a structured four-tier paradigm: preventive, mechanical, antimicrobial, and maintenance strategies. Recent studies critically emphasize the necessity of this comprehensive approach; for instance, rigorous preventive protocols focusing on tailored oral hygiene and plaque control remain fundamental to mitigating disease onset.^3^ Mechanical interventions, primarily subgingival scaling and root planing (SRP) or implant surface debridement, serve as the clinical foundation, but they often fail to completely eradicate pathogenic biofilms entrenched deep within periodontal pockets or the complex micro-porosities of implant surfaces.^4^ To augment mechanical therapy, antimicrobial strategies may involve localized or systemic administration of agents like chlorhexidine and antibiotics.^5^, ^6^ Furthermore, the adjunctive use of broad-spectrum antimicrobials carries the severe risk of inducing multidrug resistance and causing dysbiosis in the oral microbiome. Ultimately, these steps culminate in rigorous maintenance protocols, including supportive periodontal therapy (SPT) for long-term stability.^7^ Most critically, these traditional physical and chemical interventions are fundamentally incapable of precisely modulating the host immune-inflammatory response, which is the primary driver of tissue destruction and disease recurrence. To overcome these clinical bottlenecks, gene editing technology has emerged as a revolutionary tool with immense potential by direct modification of pathogenic genes.

Gene editing technology enables the precise modification of target genes within an organism—including single-nucleotide changes through base and prime editing—by inserting, replacing, or deleting specific genetic sequences, thereby obtaining desired alterations in genetic information and phenotypes.^8^ The advancement of gene editing technology has provided unprecedented opportunities for biomedical research and disease therapy. In recent years, researchers have investigated the potential application of gene editing technology for addressing human diseases, revealing great progress in treating single-gene disorders, tumors, and human mitochondrial disease, with promising outcomes.^9^, ^10^, ^11^

The first generation of gene editing technology zinc finger nucleases (ZFNs) offer distinct advantages including high efficiency and small size, and development process of this technology laid an important foundation for the subsequent advancement of transcription activator-like effector nucleases (TALENs) and clustered regularly interspaced short palindromic repeat (CRISPR)/CRISPR-associated protein (CRISPR/Cas) systems.^12^ However, this technology has some limitations, such as high costs, off-target effects and difficulties in design and operation, which limit its further application.^13^ Compared with ZFNs, the second-generation gene editing technology TALENs feature lower production costs, simplified design and operational protocols, and enhanced specificity with lower off-target effects but the coding sequence of TALE protein is longer.^14^ A major challenge for clinical translation is the efficient delivery of artificially engineered TALENs into pathological cells and the successful expression of related proteins.^12^

The CRISPR-Cas9 system relies on a customizable RNA-guided mechanism instead of laborious protein modifications, thereby achieving exceptional programmability, targeted specificity, and robust editing efficacy for precise genetic modifications.^15^ Given these superior features, the CRISPR-Cas9 system has opened new avenues for oral disease treatments, as it can effectively modify pathogenic genes to improve clinical outcomes. Although earlier reviews have broadly explored gene therapy concepts in dental medicine,^16^, ^17^ these prior accounts remain fundamentally limited in scope, depth, and translational applicability. Specifically, existing literature has predominantly focused on classical viral gene-addition strategies or broad genetic associations, while largely overlooking next-generation CRISPR modalities (e.g., base and prime editing, CRISPRa/i) and their precise epigenetic mechanisms.^18^, ^19^ Furthermore, previous overviews have introduced therapeutic applications on an unsystematic, disease-by-disease basis without establishing an integrated cross-disease synthesis, and have treated the harsh oral microenvironment (salivary degradation, dynamic pH shifts, and masticatory shear stress) merely as qualitative background rather than evaluating specific physicochemical barriers.^20^, ^21^ To bridge these critical gaps, the distinct novelty of this comprehensive review conceptualizes a unified "Prevention-Treatment-Reconstruction" paradigm paired with a dedicated diagnostic perspective; conducts a quantitative, cross-disease synthesis of editing efficacy, off-target signatures and safety risks; and critically evaluates advanced oral-tailored delivery platforms (nanoparticles, hydrogels, and fibrous membranes) to define actionable pathways for clinical translation.

Accordingly, this comprehensive narrative review is structured around three principal questions: (1) How are CRISPR-based technologies applied to address diverse oral pathologies across the prevention, treatment, reconstruction, and diagnostic spectra? (2) What advanced delivery strategies (e.g., viral vectors, nanoparticles, and biomaterials) are available to overcome physiological barriers within the oral microenvironment? and (3) What distinct advantages do these tools offer over conventional therapies, and what biosafety, ethical, and translational hurdles must be resolved before clinical implementation?

To provide a robust foundation for this narrative review, a comprehensive literature search was conducted across three major electronic databases: PubMed, Web of Science, and Scopus. The search strategy utilized exact search strings incorporating Boolean logic, such as "gene editing" OR "CRISPR" OR "CRISPR-Cas9" AND "oral diseases" OR "dental caries" OR "oral squamous cell carcinoma" OR "periodontitis" AND "delivery systems" OR "nanoparticles" OR "viral vectors". The literature selection was restricted to English-language, peer-reviewed original research and review articles published between 2010 and 2026, aligning with the inception and rapid evolution of gene editing technology. Following the removal of duplicates, articles were screened based on title and abstract relevance to the defined paradigms. The final inclusion prioritized studies that demonstrated clear molecular mechanisms, utilized robust experimental models (e.g., *in vivo* animal models or validated cell lines), or presented novel delivery platforms applicable to the oral microenvironment.

## 2 Principles of gene editing technology based on CRISPR-Cas9 system

As a form of heritable immunity, the CRISPR/Cas system equips bacteria and archaea with the ability to identify and cleave non-host DNA, providing a robust barrier against the infiltration of phages and foreign plasmids.^15^, ^22^ CRISPR sequences are stable repetitive DNA sequences composed of highly conserved repeat sequences and spacer sequences.^23^ CRISPR-RNA (crRNA), derived from the transcription of the CRISPR array, pairs with trans-activating crRNA (tracrRNA) to form a dual-RNA complex. In genetic engineering, these two RNAs are artificially fused into a synthetic single-guide RNA (sgRNA), which guides the Cas nuclease protein to precisely target and cleave specific double-stranded DNA sequences.^24^

According to the updated evolutionary classification^25^, CRISPR-Cas systems are broadly categorized into two major classes based on the architectural organization of their effector complexes, which are further divided into six distinct types (Types I–VI) and multiple subtypes. Class 1 systems (comprising Types I, III, and IV) rely on multi-subunit Cas protein complexes to execute crRNA processing and target interference, presenting operational complexity. Conversely, Class 2 systems (comprising Types II, V, and VI) are defined by a streamlined effector structure utilizing a single, multi-domain Cas protein (such as Cas9 in Type II, Cas12 in Type V, and Cas13 in Type VI). The structural minimalism of Class 2 systems confers marked advantages in terms of superior molecular programmability, therapeutic utility, editing efficiency, and functional reliability.^22^, ^26^

### 2.1 Overview of CRISPR-Cas9

CRISPR-Cas9 has established itself as the preeminent gene-editing platform. The mechanism of the CRISPR-Cas9 system can generally be categorized into three phases: acquisition of CRISPR spacer sequences, transcription and processing of CRISPR genes, and the targeted interference with foreign genes through CRISPR activity.^27^ During the initial invasion of exogenous DNA into cells, Cas protein complex identifies and cleaves a segment of exogenous DNA containing a protospacer adjacent motif (PAM) to integrate this sequence into the host CRISPR. Upon subsequent invasion of exogenous DNA, the Cas9 protein, guided by sgRNA, precisely identifies and cleaves DNA sequences containing PAM, resulting in a double-strand break (DSB).^28^ This process could trigger innate DNA repair mechanisms, including non-homologous end joining (NHEJ) or homology-directed repair (HDR), ultimately achieving gene editing.^29^ (Fig. 1). The inherent inaccuracy of NHEJ typically yields frameshift mutations, making it a highly efficient strategy for gene ablation. Conversely, HDR depends on a supplied donor sequence to orchestrate high-fidelity genetic edits, such as tailor-made insertions or precise allelic replacements.^30^, ^31^ Crucially, editing efficiency is highly cell-type dependent within the oral cavity. For instance, CRISPR-Cas9 knockout efficiencies can reach up to 70-80% in oral squamous cell carcinoma (OSCC) cell lines (e.g., CAL27) and human gingival fibroblasts (HGF-1) cells under optimized electroporation or viral transduction.^32^, ^33^ In contrast, editing efficiency in primary oral epithelial cells or recalcitrant bacterial species like *S. mutans* is often significantly lower (typically 10-30%) due to robust physical barriers and lower transformation rates, necessitating specific promoter optimizations and carrier enhancements.^34^, ^35^

**Fig. 1:**
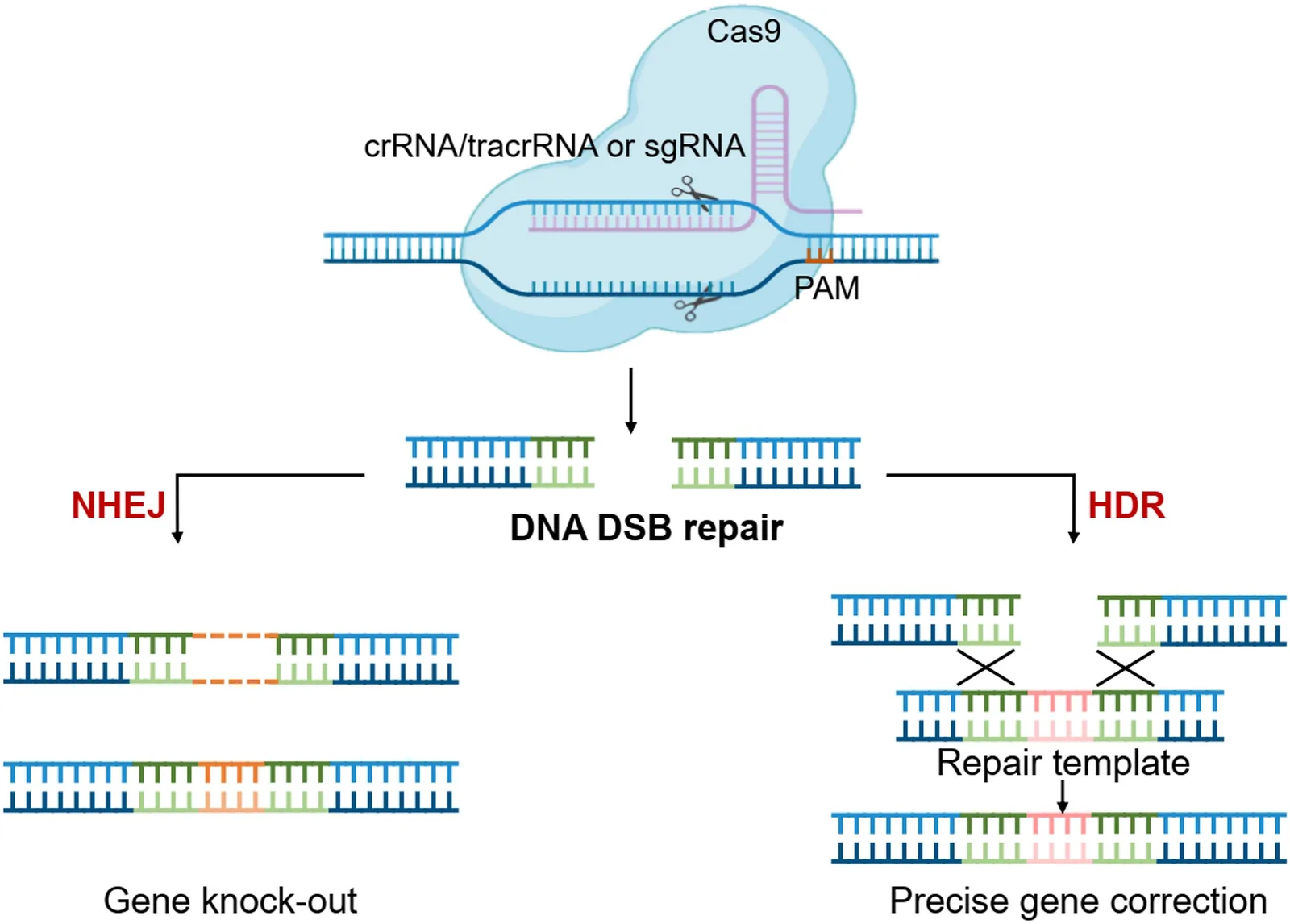
Molecular principles of gene editing based on CRISPR-Cas9. The Cas9 endonuclease, guided by a dual-RNA complex consisting of crRNA and tracrRNA or a sgRNA, precisely targets specific DNA sequences. Target recognition requires two critical elements: sequence complementarity between the guide RNA's spacer region and the target DNA, along with the presence of a PAM immediately downstream of the target sequence. Upon successful binding, the Cas9 enzyme induces a DSB at the target site through its nuclease activity. This DNA break subsequently activates cellular intrinsic repair pathways, primarily NHEJ or HDR.

The development of gene editing technology, especially the application of the CRISPR-Cas9 system, holds immense promise for fundamental research and clinical treatment. CRISPR-Cas9 has been harnessed to reprogram Chimeric Antigen Receptor T-cells (CAR-T) through the site-specific knock-in of a CD19-targeted CAR construct into the endogenous T-cell receptor α constant (*TRAC*) locus. This strategic modification significantly mitigates T-cell exhaustion and preserves a less-differentiated state, thereby potentiating the durable anti-tumor efficacy of CAR-T in the engineered cells.^31^, ^36^ The application of an adeno-associated virus (AAV) vector for the targeted delivery of the CRISPR system to photoreceptor cells allowed for the precise excision of the aberrant exon donor site associated with the IVS26 mutation, while maintaining the functional integrity of the wild-type allele. This strategy successfully restored physiologically normal CEP290 protein expression, demonstrating a therapeutic potential in Leber's congenital amaurosis type 10 (LCA10).^37^ Recently, CRISPR-based editing of simian immunodeficiency virus (SIV) proviral DNA in ART-treated primates *in vivo* has been reported.^38^ In contrast to conventional anti-retroviral treatments alone, this gene-editing strategy effectively disrupted the viral genome through multiple targeted cleavage events, signifying a crucial preclinical step towards a functional cure for related retroviral diseases such as AIDS. In addition, CRISPR-Cas9 has made significant progress in the research and treatment of various diseases, such as phenylketonuria^39^, transfusion-dependent β-thalassemia^40^, and primary mitochondrial disorders^11^, which provides valuable implications for the treatment of oral diseases.

### 2.2 Next-generation CRISPR modalities and precision tools

Beyond conventional Cas9-mediated double-strand breaks (DSBs), relying exclusively on nucleases poses intrinsic risks of uncontrolled insertions/deletions (indels), genomic translocations, and DNA damage responses. To circumvent these limitations, the CRISPR toolbox has expanded to include precise, non-cleavage, and regulatory modalities (Table 1).

**Table 1:** Comprehensive comparison of novel CRISPR-based gene editing and regulatory modalities

Technology	Mechanism	Cargo requirements	Permanence	Efficiency	Safety/Off-target	Ref
Conventional Cas9 (DSB)	Target DNA cleavage resulting in DSBs repaired via NHEJ/HDR	Large (∼4.1 kb SpCas9 + guide RNA); near single AAV capacity limit	Permanent	High for gene knockout (NHEJ); variable for knock-in (HDR)	Moderate; potential for translocations, large deletions, and p53 activation	51, 52, 53
Base Editing (BE)	nCas9 + Cytidine/ Adenine Deaminase; converting targeted base pairs (C·G to T·A, or A·T to G·C)	Very Large (∼5.0–5.5 kb); requiring dual-AAV, mRNA, or LNP delivery	Permanent	High for point mutations; limited by bystander editing window	High; no DSBs, low indel rates; potential RNA/DNA off-target deamination	41, 42, 54
Prime Editing (PE)	nCas9 + reverse transcriptase + pegRNA; copying template sequence into target locus	Very large (>6.0 kb); exceeding single AAV limits	Permanent	Moderate to High; depending on pegRNA structural design	Very High; minimal off-target editing, no DSB requirement	^43^
CRISPRi / CRISPRa	dCas9 + transcriptional Repressor (KRAB) or Activator (VP64/VPR)	Large (dCas9 + effector domains)	Reversible; requiring continuous vector expression	High for transcriptional activation or silencing	High; no DNA cleavage, minimal permanent off-target toxicity	44, 55
Epigenome Editing	dCas9 + Epigenetic modifier (e.g., DNMT, TET, HAT, HDAC)	Large (dCas9 + modifying domain)	Semi-permanent / Mitotically heritable	Variable; locus-dependent chromatin remodeling	High; modulating gene activity without genomic DNA modification	45, 46
RNA Targeting (Cas13)	Type VI Cas13; RNA-guided single-stranded RNA transcript degradation	Moderate (∼3.0–3.5 kb); easily packaged within single AAV	Transient / Reversible	High for post-transcriptional knockdown	High; no host genomic integration; requiring monitoring collateral RNA cleavage	47, 48
Compact Cas Systems	Miniature effectors (e.g., Cas12f, CasPhi, CasMINI) targeting DNA/RNA	Small (<1.5–2.5 kb); ideal for all-in-one AAV packaging	Permanent / Variable depending on effector	Moderate to High; continuously optimizing	High; reduced steric hindrance, improved specificity	49, 50

Specifically, Base Editing (BE) and Prime Editing (PE) facilitate exact nucleotide substitutions and targeted sequence modifications without requiring DSBs or donor DNA templates, thereby significantly reducing off-target genomic toxicity.^41^, ^42^, ^43^ For therapeutic applications requiring gene modulation rather than irreversible sequence alteration, CRISPR interference/activation (CRISPRi/a) and epigenome editors offer programmable, locus-specific transcriptional control.^44^, ^45^, ^46^ Furthermore, RNA-targeting endonucleases like Cas13 provide a transient and reversible alternative to DNA editing.^47^, ^48^ To overcome strict packaging capacity of adeno-associated virus (AAV) vectors, the discovery and engineering of hypercompact systems, such as CasPhi and CasMINI, have greatly enhanced the feasibility of *in vivo* delivery.^49^, ^50^

## 3 Relationship between gene editing and oral diseases

Crucially, the oral cavity presents a uniquely advantageous environment for translating these gene editing tools into clinical practice: its open accessibility allows for precise, localized administration of gene-editing agents, thereby maximizing therapeutic efficacy while circumventing the severe risks of systemic toxicity. Rather than merely cataloging isolated applications, current research demonstrates that gene editing shows potential to establish a comprehensive "prevention, treatment, and reconstruction" paradigm across distinct oral conditions. Specifically, this technology shows potential in the prevention of dental caries through precision microbiome remodeling, provides a proof-of-concept for the treatment of periodontitis and oral squamous cell carcinoma (OSCC) via host immune modulation and targeted cell killing, and might serve as a future strategy for the functional reconstruction of maxillofacial bone defects through tissue regeneration. Consequently, gene editing provides a multidimensional strategy for advancing oral healthcare (Fig. 2).

**Fig. 2:**
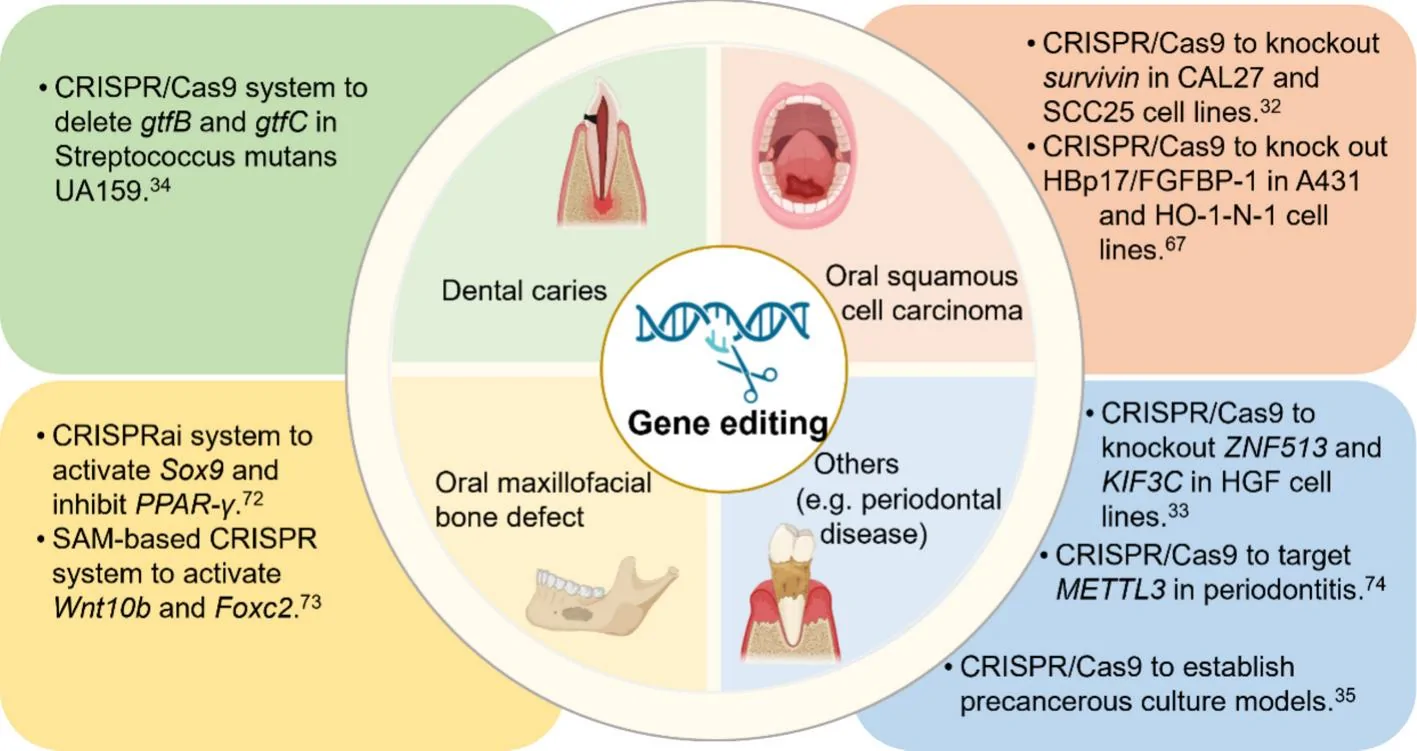
The application of gene editing technology in the fields of oral diseases, including dental caries, oral squamous cell carcinoma (OSCC), oral and maxillofacial bone defects and others.

### 3.1 The prevention paradigm: Precision microbiome remodeling in dental caries

As a chronic oral infectious disease, dental caries causes irreversible destruction of dental hard tissues and has been designated by the World Health Organization (WHO) as a priority disease for prevention and control.^56^ While conventional clinical management ultimately relies on restorative procedures for established lesions, the advent of CRISPR-based interventions introduces a profound shift toward a prevention paradigm. By directly targeting the root causes of cariogenic dysbiosis, gene editing aims to intercept disease progression before irreversible damage occurs. The etiology of dental caries involves complex interactions among microflora, dietary substrate and host susceptibility, with pathogenic bacteria playing the central biological role.^57^ Current clinical management primarily relies on conventional restorative procedures and non-specific broad-spectrum antimicrobials, which fail to selectively target cariogenic pathogens or prevent disease recurrence. Therefore, developing targeted molecular therapies remains an urgent priority.

According to the ecological plaque hypothesis, dental caries is driven by a polymicrobial dysbiosis rather than infection by a single pathogen, where environmental shifts favor the overgrowth of acidogenic and aciduric species. Within this complex ecological network, *Streptococcus mutans* (*S. mutans*) acts as a keystone species orchestrating cariogenic dysbiosis, largely due to its acid production, acid tolerance, extracellular polysaccharide (EPS) synthesis, and biofilm formation.^58^, ^59^ While traditional gene editing in *S. mutans* was initially constrained by low transformation efficiency and limited genetic tools^60^, recent advances in CRISPR technology have enabled precise gene targeting. For instance, a self-targeting gene editing system was developed by utilizing the endogenous CRISPR-Cas9 machinery of *S. mutans* UA159. By successfully deleting the *gtfB* and *gtfC* genes, which encode glucosyltransferases responsible for EPS synthesis, biofilm formation and virulence were markedly impaired without disrupting the overall microbial ecosystem in bacterial models^34^ (Fig. 3). This targeted disruption of specific virulence genes revealed the potential of gene editing as a promising strategy for oral caries prevention and management.

**Fig. 3:**
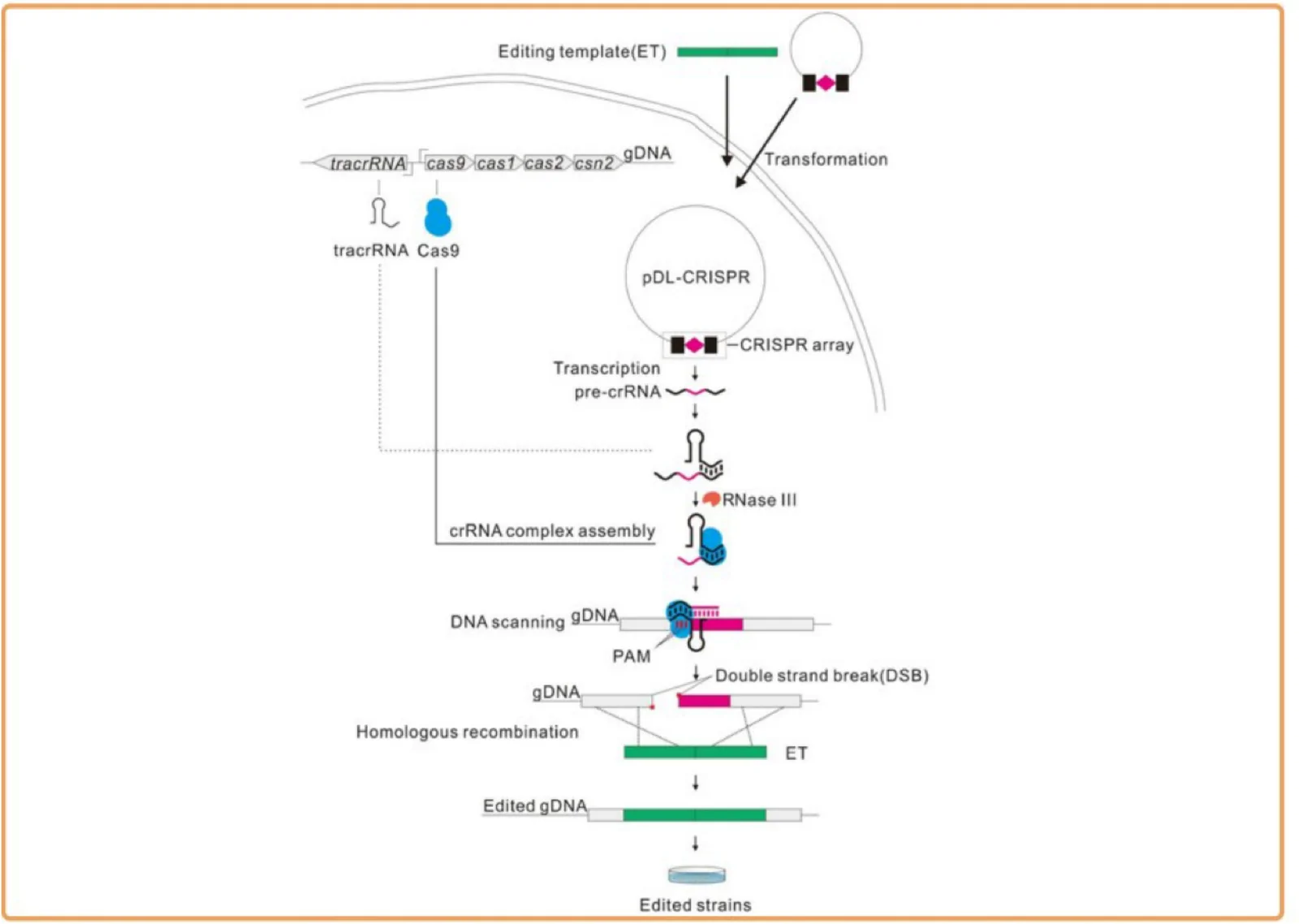
Application of gene editing in the treatment of oral caries. The four stages of the CRISPR-Cas interference pathway: (a) transformation stage. Plasmids and editing templates are cotransformed into Streptococcus mutans UA159 using competence-stimulating peptide (CSP) to enhance transformation efficiency; (b) crRNA biogenesis stage. The CRISPR array is transcribed from the vector, while the tracrRNA precursor, Cas9, and RNase III are expressed from the genome. pre-crRNAs are subsequently processed into mature crRNAs by RNase III and other factors; (c) Target interference stage. crRNAs assemble with Cas proteins into effector complexes, which use these RNAs as guides to locate and cleave complementary target sequences, generating double-strand breaks (DSBs); (d) Editing stage. Homologous recombination repair is initiated by DSBs using editing templates.^34^ (Adapted with permission of ref.^34^ Copyright © 2018 John Wiley and Sons.)

### 3.2 The treatment paradigm: Targeted gene editing in oral squamous cell carcinoma

Oral cancer is one of the most common multiple malignant tumors in the world accounting for approximately 3% of all global malignant tumors, which poses a serious threat to human life and health.^61^ Given its aggressive nature and high metastatic potential, gene editing in OSCC has been predominantly explored as a therapeutic strategy. Unlike early screening or preventive measures, current CRISPR technologies are being explicitly harnessed to develop targeted molecular therapeutics that directly inhibit tumor progression. Although important strategies have been explored in cancer treatment, the mortality rate of oral cancer has not decreased since the 1960s, and the 5-year survival rate remains at about 50%.^62^ Among oral cancer, OSCC accounts for up to 90% and is the most common type of oral cancer.^63^

The main treatment for OSCC is surgery, radiotherapy and chemotherapy. The occurrence location of OSCC is surrounded by abundant vascularization and complex lymphatic networks, so OSCC is prone to metastasis and has a poor clinical prognosis.^64^ In addition, surgical treatments can cause varying degrees of damage to the patient's appearance, leading to certain psychological impacts for patients. Radiotherapy often leads to a spectrum of adverse effects, including pain, fatigue, dry mouth, and anorexia, significantly impairing patients' quality of life.^65^ Chemotherapy drugs inevitably have toxic side effects that harm normal body tissues.^66^ Therefore, it is of great significance to increase survival rate and improve prognostic life of patients by understanding pathogenesis and finding new therapeutic methods. Li et al. found that the apoptosis inhibitor gene *survivin* was highly expressed in OSCC tumor tissues and cells.^32^ Using CRISPR-Cas9-based gene editing technology, the *survivin*-knockout CAL27 and SCC25 cell lines were constructed for *in vitro* assays and *in vivo* murine xenograft models. The results showed a marked suppression in cell viability, encompassing reduced cell proliferation, impeded colony formation *in vitro*, and inhibited tumor growth *in vivo*. This provides a proof-of-concept that the knockout of *survivin* might serve as a therapeutic strategy for OSCC (Fig. 4a). Shintani et al. used CRISPR-Cas9 technology to knock out *HBp17/FGFBP-1* in A431 and HO-1-N-1 cell lines.^67^ Results indicated that cell proliferation, colony formation, and cell motility *in vitro*, as well as tumor growth in murine xenograft models, were significantly inhibited, suggesting *HBp17/FGFBP-1* as a potential therapeutic target for OSCC (Fig. 4b). However, extensive clinical validation is required to translate these preclinical findings into safe and effective human therapeutics.

**Fig. 4:**
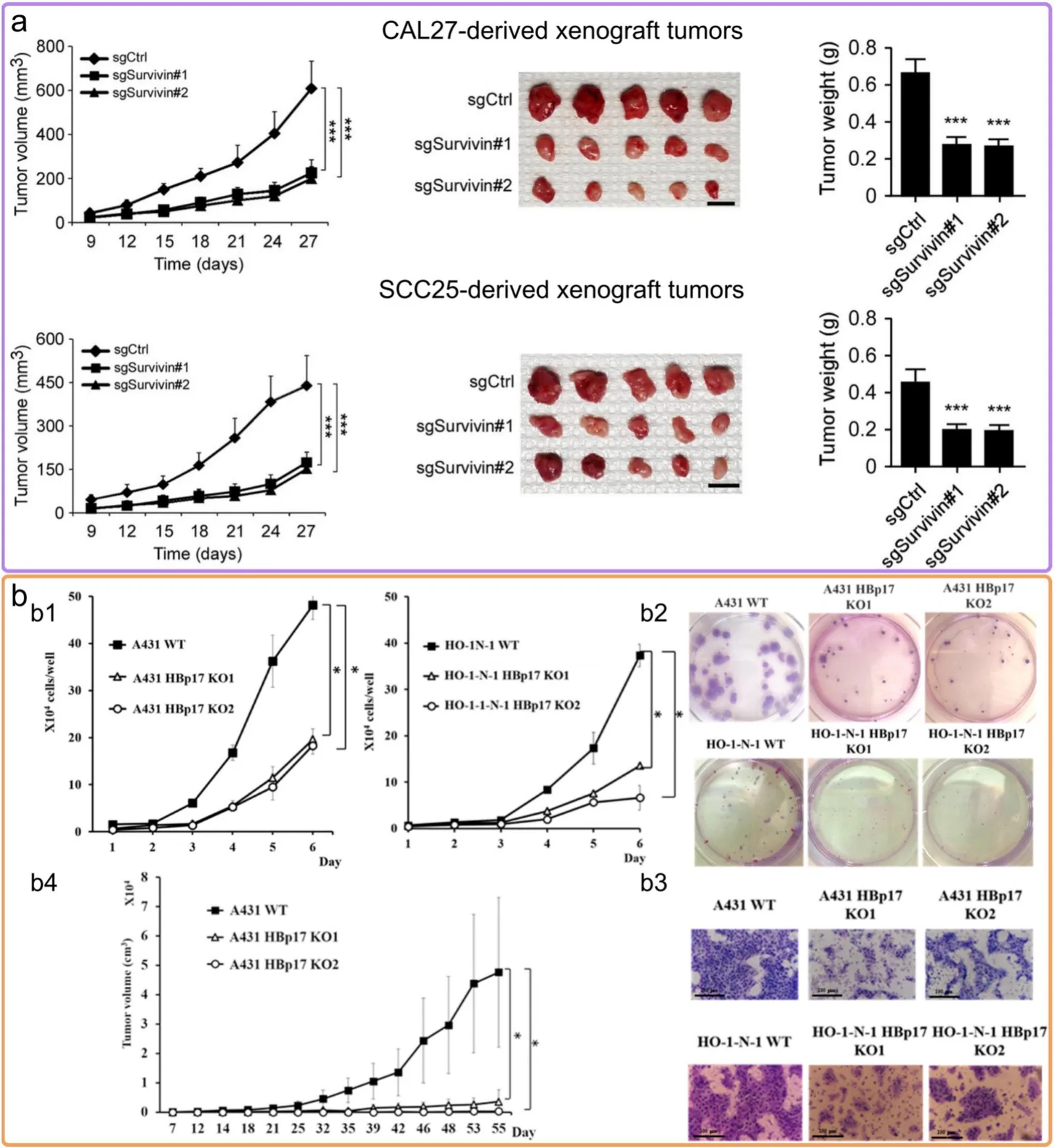
Application of gene editing in the treatment of OSCC. a. The tumor volume, the image of tumor mass, and tumor weight of CAL27-derived or SCC25-derived xenograft tumors with *survivin* knockout or not.^32^ (Fig. 4a was adapted with permission of ref.^32^ Copyright © 2020 BioMed Central.) b. The cell proliferation (b1), colony formation (b2), cell mobility (b3) and tumor growth (b4) evaluated by knockout of HBp17.^67^ (Fig. 4b was adapted with permission of ref.^67^ Copyright © 2021 MDPI.)

### 3.3 The reconstruction paradigm: Gene editing for oral and maxillofacial bone regeneration

In the context of severe hard tissue loss, therapeutic efforts transition inherently into a reconstructive approach. Here, rather than being limited to disease prevention or targeted cell killing, gene editing is increasingly directed toward orchestrating cellular reprogramming for tissue regeneration. The oral and maxillofacial region comprises a complex arrangement of multiple bones, whose structural integrity forms the essential foundation for maintaining normal anatomical morphology and physiological function of the maxillofacial region.^68^ However, the causes of maxillofacial bone injury are diverse, including trauma, infection, tumor resection, and congenital malformation.^69^ Bone defects not only affect the patient's appearance but also impair their speech, chewing and other functions.^68^ In particular, the maxilla and mandible, which are important supporting structures of the face, can have particularly severe impacts on patients when they are defective.

Traditional bone repair materials mainly include autogenous bone, allograft bone, and xenograft bone, but these methods all have certain limitations. Although autogenous bones have good biocompatibility, some drawbacks, such as limited bone harvest, infection, or tissue damage in the donor area still exist.^70^ Allograft bones and xenograft bones may cause problems such as immune rejection and disease transmission.^71^ The combined application of CRISPR-Cas9-mediated gene editing and tissue engineering shows potential in promoting bone repair. Truong et al. established a CRISPRai system consisting of inactive Cas9, fusion proteins, and sgRNA. This system was subsequently packaged into an all-in-one baculovirus vector for efficient delivery to rat bone marrow mesenchymal stem cells (rBMSCs), which simultaneously activated *Sox9* and inhibited *PPAR-γ*.^72^ Activation of *Sox9* promoted chondrogenesis of rBMSC, while inhibition of *PPAR-γ* repressed adipogenesis of rBMSC. Implantation of rBMSCs engineered by the CRISPRai improved calvarial bone healing in rat calvarial-defect models, providing therapeutic potentials for bone regeneration (Fig. 5a). Hsu et al. employed an advanced hybrid baculovirus vector system for efficient gene delivery and exploited a synergistic activation mediator (SAM)-based CRISPR system into rat BMSCs to activate *Wnt10b* and *Foxc2*.^73^ Individual activation of either *Wnt10b* or *Foxc2* can enhance osteogenesis while inhibiting adipogenesis *in vitro*. The synergistic activation of both *Wnt10b* and *Foxc2* potentiated the osteogenic differentiation of BMSCs *in vitro* and markedly improved calvarial bone healing *in vivo* in rats. This showed the promising potential of CRISPRa technology for applications in bone tissue regeneration (Fig. 5b). However, these results are currently limited to animal or cellular models, and there is a long way to go as their feasibility, safety, and comparative efficacy against existing therapies have yet to be demonstrated in human subjects.

**Fig. 5:**
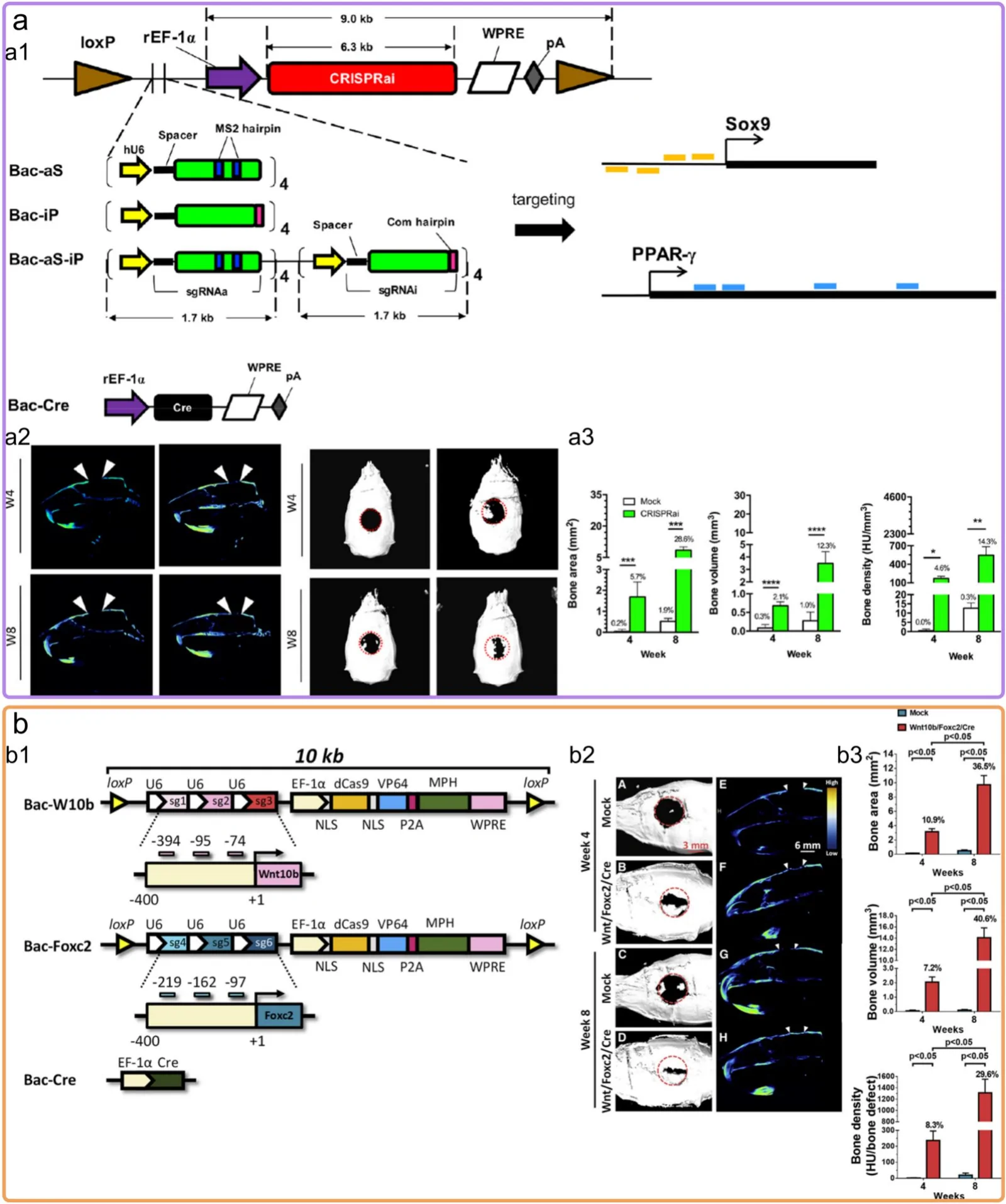
Application of gene editing in the treatment of bone defect. a. CRISPRai delivered by baculovirus activated *Sox9* and repressed *PPAR-γ* for bone regeneration.^72^ (a1) Baculovirus vectors and targeting sites. BacaS-iP expressed four *Sox9*-targeting sgRNAa and four *PPAR-γ*-targeting sgRNAi. (a2) The sagittal and top views from *in vivo* bone healing evaluated by μCT imaging. (a3) Quantitative analysis of bone area, volume and density. (Fig. 5a was adapted with permission of ref.^72^ Copyright © 2019 Oxford University Press.) b. Construction of baculoviruses for *Wnt10b* and *Foxc2* activation for bone regeneration.^73^ (b1) Schematic of Bac-W10b, Bac-*Foxc2*, and Bac-Cre vectors. (b2) The top views and sagittal views of regenerated bones at weeks 4 and 8. (b3) Quantitative analysis of bone area, volume and density. (Fig. 5b was adapted with permission of ref.^73^ Copyright © 2020 Elsevier.)

### 3.4 Extending the paradigm: Relationship between gene editing and other oral diseases

Beyond the core paradigms of prevention, treatment, and reconstruction, gene editing serves as a highly versatile platform for elucidating complex pathogenic mechanisms across a broader spectrum of oral conditions. Gene editing technology further offers research methodologies for exploring additional oral diseases. To investigate human gingival fibromatosis (HGF), CRISPR-Cas9 gene editing was employed to create *ZNF513*-knockout and *KIF3C*-knockout *in vitro* human gingival fibroblast (HGF-1) cell lines, thereby elucidating the roles and interactions of *ZNF513* and *KIF3C* in the pathogenesis of HGF.^33^ Additionally, CRISPR-Cas9 systems were utilized to investigate the *METTL3* gene, clarifying its regulatory role in promoting NLRP3 inflammasome activation and the subsequent progression of periodontitis.^74^ Furthermore, CRISPR-Cas9 editing facilitated the establishment of precancerous culture models for oral keratinocyte cultures, enabling the assessment of the effectiveness of a panel of small molecule inhibitors.^35^

### 3.5 Complementary perspectives: CRISPR-based molecular diagnostics in oral diseases

Beyond therapeutic gene editing, CRISPR-Cas systems—particularly Class 2 Type VI (Cas13) and Type V (Cas12) effectors—have revolutionized molecular diagnostics through their target-activated, non-specific "collateral cleavage" activity.^75^ While therapeutic gene editing faces substantial *in vivo* delivery hurdles and biosafety concerns, CRISPR-based diagnostics present an immediate, non-invasive translational modality that is likely to enter routine clinical use far sooner.^76^

In oral healthcare, conventional microbial assays such as quantitative PCR (qPCR) and next-generation sequencing (NGS) are often constrained by prolonged turnaround times, high operational costs, and technical complexity.^77^ To address these clinical bottlenecks, CRISPR-powered nucleic acid assays have emerged as rapid point-of-care (POC) alternatives. ^78^ Notably, Liu et al. established a one-pot nucleic acid assay termed Specific High-Sensitivity Enzymatic Reporter UnLOCKing (SHERLOCK) by harnessing the RNA-guided RNase activity of CRISPR-Cas13a. By coupling recombinase polymerase amplification (RPA) primers with species-specific crRNAs targeting variable genetic regions, this platform enabled ultra-sensitive, single-nucleotide discrimination of oral pathogens. Crucially, when validated on unpurified saliva specimens collected from 30 healthy human donors, the SHERLOCK assay demonstrated 100% concordance with gold-standard 16S rRNA sequencing within 30 to 60 minutes. This rapid progress and high expandability highlight the practical viability of saliva-based CRISPR diagnostics.^79^

Furthermore, CRISPR-driven diagnostics hold profound translational prospects across the entire spectrum of oral pathologies by enabling multifaceted molecular detection. In caries management, the ultra-sensitive identification of cariogenic pathogens (such as *Streptococcus mutans*) facilitates early risk stratification and proactive screening of high-risk populations prior to irreversible enamel cavitation.^56^ Simultaneously, multiplexed CRISPR assays permit rapid on-site mapping of dysbiotic subgingival pathogens (e.g., *Porphyromonas gingivalis* and *Treponema denticola*), which guides personalized supportive periodontal therapy.^3^ Beyond microbial profiling, CRISPR-based biosensing can be extended to detect low-abundance circulating tumor DNA (ctDNA), specific oncogenic mutations (e.g., TP53 or NOTCH1), and aberrant salivary microRNA signatures in OSCC, offering a non-invasive liquid biopsy strategy for early tumor screening, precise molecular subtyping, and longitudinal surveillance of malignant transformation.^80^

### 3.6 Cross-disease Synthesis: Comparative parameters and translational bottlenecks

While gene editing offers versatile solutions across a spectrum of oral diseases, comparative analyses reveal highly distinct translational directions dictated by the specific pathology (Table 2). Importantly, a critical appraisal of the literature underscores that therapeutic editing remains largely preclinical, with human clinical evidence exceedingly scarce, CRISPR-based diagnostic tools present a substantially shorter translational timeline toward immediate clinical utilization.

**Table 2:** Comprehensive Summary of Parameters in CRISPR-Based Applications Across Oral Diseases

Evaluation Parameter	Dental Caries (Prevention Paradigm)	OSCC (Treatment Paradigm)	Bone Defects (Reconstruction Paradigm)
Target Gene	*gtfB*, *gtfC*	*survivin*, *HBp17*	*Sox9*, *PPAR-γ*, *Wnt10b*, *Foxc2*
Editing System	Endogenous CRISPR-Cas9	CRISPR-Cas9	CRISPRai/SAM-CRISPRa
Cargo Format	Plasmid	Plasmid/Viral vectors	All-in-one baculovirus vector
Delivery Route	Cotransformation (*in vitro*)	Transfection/Intratumoral injection	*Ex vivo* transduction & local implantation
Experimental Model	*S. mutans* UA159 bacterial strains	CAL27, SCC25, A431, HO-1-N-1 cells; murine xenograft models	rBMSCs; rat calvarial-defect models
Evidence Stage	Preclinical (*in vitro*)	Preclinical (*in vivo*)	Preclinical (*in vivo*)
Editing Efficiency	High targeted deletion rate	High gene knockout efficiency	Robust multiplexed gene activation
Biological Outcome	Marked impairment of biofilm formation and bacterial virulence	Significant suppression of tumor cell viability and *in vivo* tumor growth	Enhanced osteogenesis and accelerated calvarial bone healing
Off-target Assessment	Low off-target effects mentioned	Limited genome-wide screening available	Minimal off-target transcription observed
Safety Risks	Horizontal gene transfer; ecological microbiome disruption	Off-target somatic mutations; immune responses	Vector immunogenicity; potential insertional mutagenesis
Translational Potential	Moderate	High for localized delivery	High for scaffold-based tissue engineering
Key Bottleneck	Requires pH-responsive and saliva-resistant vectors for biofilm penetration	Overcoming dense tumor stroma; avoiding systemic off-target toxicity	Maintaining sustained vector release under masticatory mechanical stress
References	34	32,67	72,73

Through cross-disease synthesis, clear patterns emerge regarding editing efficiency, delivery difficulty, and safety profiles. Regarding editing efficiency, ablative CRISPR-Cas9 interventions targeting single virulence genes in bacteria or anti-apoptotic genes in tumors generally exhibit high immediate knockout efficacy.^32^, ^34^, ^67^ In contrast, bone reconstruction demands complex transcriptional modulation to orchestrate multigenic osteogenic pathways, posing a higher efficiency barrier.^72^, ^73^ Furthermore, delivery difficulty varies significantly across the oral landscape: caries prevention necessitates nanocarriers capable of penetrating dense extracellular polymeric substance (EPS) biofilms while resisting salivary washout;^58^ OSCC treatment faces the biological barriers of dense tumor stroma and lymphatic networks;^64^ whereas maxillofacial reconstruction requires sustained, localized vector release under continuous masticatory mechanical stress.^68^ In terms of safety and off-target effects, the risks are highly context-dependent. Microbiome remodeling in caries primarily risks environmental horizontal gene transfer within the dysbiotic flora, contrasting sharply with OSCC and bone reconstruction, where off-target somatic mutations, chromosomal translocations, and vector-induced immunogenicity are the paramount concerns.^29^, ^53^, ^81^ Ultimately, the shared research gaps and bottlenecks across all paradigms remain the lack of *in vivo* safety data in human subjects, coupled with the urgent need for oral-environment-responsive smart delivery carriers to bridge the gap between bench bridge bench-to-bedside translation.^82^, ^83^

## 4 Delivery methods using gene editors to treat oral diseases

Due to the complexity and diversity of oral diseases, gene editing tools not only need to ensure the effectiveness and specificity of treatments, but also need to select the appropriate delivery methods according to the different features. More importantly, the unique and challenging microenvironment of the oral cavity was characterized by continuous salivary washout, complex polymicrobial biofilms, and mechanical forces. Specifically, the oral pH typically fluctuates between 6.2 and 7.4; acidic environments (pH approaching 6.2), frequently exacerbated by cariogenic biofilms, can induce protonation of key amino acid residues in the Cas9 protein, thereby altering its conformational stability and significantly reducing its endonuclease cleavage activity.^84^ Furthermore, human saliva contains abundant nucleases and proteolytic enzymes (such as matrix metalloproteinases and amylases), which can rapidly degrade naked guide RNAs and cleave Cas proteins through enzymatic hydrolysis, severely shortening their half-life.^85^ Crucially, these salivary components also directly compromise the structural integrity of delivery vectors. For instance, salivary lipases and surfactants can destabilize the lipid bilayers of liposomes and lipid nanoparticles (LNPs) leading to premature cargo leakage, while salivary proteases can denature viral vector surface glycoproteins, thereby neutralizing their transduction capabilities.^86^ This makes the surface engineering of delivery carriers essential for preserving the conformational stability and bioavailability of gene-editing tools. For example, surface PEGylation or zwitterionic polymer functionalization establishes a dense steric hydration layer that minimizes non-specific salivary mucin adsorption and prevents enzymatic degradation.^87^ Furthermore, incorporating proton-sponge nanocarriers and pH-responsive charge-reversal polymers, such as 2-(dimethylamino)ethyl methacrylate or histidine-rich motifs, effectively buffers against acidic protonation within cariogenic niches while promoting endosomal escape.^88^ In addition, bio-responsive protective shells inhibit premature carrier disintegration in salivary fluids while enabling localized release at pathological sites.^89^ At present, the delivery methods are mainly divided into various categories: physical manners, viral vectors, nanoparticle carriers and others (Table 3).

**Table 3:** Comprehensive evaluation of delivery strategies for CRISPR-Cas systems

Delivery Vehicle (Class)	Cargo / Capacity	Expression / Delivery Strategy / Targeting	Safety profile (Immunogenicity & Insertional risk)	Evidence in oral applications (Refs)
Physical manners	DNA, mRNA, RNP Capacity: Unlimited	Transient expression; primarily *ex vivo*; poor targeting	Immunogenicity: Low Insertional risk: None	None reported yet for CRISPR^90^, ^91^
Lentivirus (Viral vectors)	DNA (via RNA genome) Capacity: ∼8 kilobases limit	Persistent expression; primarily *ex vivo*; moderate targeting	Immunogenicity: Moderate Insertional risk: High	None reported yet for CRISPR^92^, ^93^
Adenovirus (Viral vectors)	DNA Capacity: Large (up to 36 kilobases)	Transient to persistent; *ex vivo* and *in vivo*; moderate targeting	Immunogenicity: High Insertional risk: Low	None reported yet for CRISPR^92^, ^94^
Adeno-associated virus (AAV) (Viral vectors)	DNA Capacity: Low (∼4.7 kilobases limit)	Persistent expression; primarily *in vivo*; high targeting	Immunogenicity: Low to Moderate Insertional risk: Low	None reported yet for CRISPR^92^, ^95^
Baculovirus (Viral vectors)	DNA Capacity: Very large (>30 kilobases)	Transient to persistent; *ex vivo* and *in vivo*; high targeting	Immunogenicity: Moderate Insertional risk: Low	Delivery of CRISPRa for rat calvarial bone regeneration 72, 73
Lipid nanoparticle (LNP) (Nanoparticles carriers)	DNA, mRNA, RNP Capacity: Large	Transient expression; highly scalable *in vivo*; moderate to High targeting	Immunogenicity: Low Insertional risk: None	Delivery of HuR CRISPR targeting OSCC *in vivo*^96^
Gold nanoparticle (Nanoparticles carriers)	DNA, mRNA, RNP Capacity: Moderate	Transient expression; *ex vivo* and *in vivo*; high targeting	Immunogenicity: Low Insertional risk: None	None reported yet for CRISPR^97^, ^98^
Nanopolymer (Nanoparticles carriers)	DNA, mRNA, RNP Capacity: Large	Transient expression; *ex vivo* and *in vivo*; moderate targeting	Immunogenicity: Low Insertional risk: None	None reported yet for CRISPR^99^, ^100^
Macroscopic Biomaterials (Hydrogels and Fibers)	DNA, RNP (encapsulated) Capacity: Very large	Sustained transient release; *in vivo* (local implantation); localized targeting	Immunogenicity: Low Insertional risk: None	None reported yet for CRISPR^101^, ^102^

### 4.1 Physical manners

Physical mediation mainly includes electroporation^103^, microinjection^104^, hydrodynamic injection^105^, microfluidic membrane deformation^106^, etc. Electroporation is the most commonly used method. In 1982, Neumann first proposed the electric impulse method as a simple, readily applicable, and highly efficient way to transfer linear or circular DNA into recipient cells, resulting in successful gene expression.^90^ The underlying principle of electroporation is to create perforations in the cell membrane by applying electric fields, allowing DNA, RNA, proteins, and other exogenous materials to enter cells.^91^ Electroporation has evolved as a prevalent physical delivery method due to its advantages of high delivery efficiency and ability to deliver multiple substances. Sato et al. directly injected a plasmid expressing green fluorescent protein (GFP) into the pancreas of adult anesthetized female mice and performed electroporation using a square pulse generator after wound closure.^107^ One day post-gene delivery, successful expression of GFP was observed at the injection site, with the GFP expression level reverting to baseline within a week of transfection. This suggested that electroporation can be safely and efficiently employed for transfecting pancreatic cells. The combination of electroporation and the CRISPR-Cas9 system for gene editing shows remarkable potential. However, the development of electroporation is limited because of its application difficulty *in vivo*, the formation of uneven aperture, and mechanical damage.^91^ Overall, electroporation presents a promising delivery method with significant potential for application in oral disease treatment.

### 4.2 Viral vectors

Viral vectors such as lentiviruses, adenoviruses and AAV are widely used in CRISPR system delivery. Lentivirus is a double-stranded RNA virus with reverse transcription and integration functions, enabling the stable incorporation and expression of foreign genes within the host cell.^92^, ^108^ Lentivirus has a large load capacity, high transfection efficiency, and can infect non-dividing and dividing cells.^93^ Aiuti et al. employed lentiviruses carrying functional WASP genes to genetically modify hematopoietic stem cells (HSCs) derived from three patients with Wiskott-Aldrich syndrome (WAS).^109^ WASP-expressing cells of all patients showed stable transplantation, and the platelet count, immune function and clinical score were improved after cell infusion. However, the integration of lentiviral DNA into the host genome carries the risk of random insertional mutations, potentially disrupting cellular genotypes and posing safety challenges, thereby limiting their therapeutic application.^92^

Adenovirus is a double-stranded linear DNA virus. Adenoviral vectors have a large loading capacity and are capable of delivering exogenous genes without incorporating them into the host genome, thereby reducing the risk of insertional mutagenesis.^94^ Wu et al. designed a genetically modified adenovirus vector to deliver the CRISPR-Cas9 system (sgCas9-AdV) for permanent *PD-L1* gene editing in Hepa 1-6 liver cancer cells.^110^ The system revealed good tumor inhibition effects in multiple models. Nevertheless, the expression of exogenous genes delivered by adenovirus will weaken over time. Due to its pathogenicity, the introduction of adenovirus vectors can trigger the body’s immune response.^92^, ^108^

AAVs are single-stranded DNA viruses that rely on helper viruses to provide the necessary replication enzymes for replication.^111^ Compared to adenoviruses, AAVs provide enhanced safety, reduced immunogenicity, and the capacity for sustained expression of exogenous genes. However, AAVs show low load capacity.^92^, ^108^ Currently, there are different measures to improve the loading ratio. One is to design smaller Cas proteins, and the other is to use 2 or 3 AAVs to bind gene components for delivery, but the transduction efficiency of the latter is significantly lower than that of the former.^95^ Studies had successfully generated enAsCas12f protein in the CRISPR-Cas12f system, which was about one-third the size of SpCas9. The enAsCas12f protein had good activity and a low off-target rate. When combined with exogenous genes and delivered to mice using AAV vectors, the system demonstrated efficient knock-in/knock-out activity and transcriptional activation capabilities. This approach enhanced the effective payload capacity of the delivery system.^112^ So, the use of AAVs to deliver CRISPR systems to treat oral diseases is important.

### 4.3 Nanoparticles carriers

A number of nonviral vectors are developed to enhance delivery efficiency, minimize potential immunogenicity, and simplify production processes.^113^ Currently, nanocarriers such as lipid nanoparticles (LNP), gold nanoparticles and nanopolymers have been gradually applied in the field of biomedicine.

LNPs serve as a reliable delivery platform for the CRISPR-Cas9 system, ensuring the safe and efficient transport of its components into the body.^114^ LNP can utilize the electrostatic interaction between negatively charged sgRNA and positively charged lipid head to form a complex of CRISPR-Cas9 system by lipid encapsulation. Protected by the lipid layer, the complex can enter the body via cellular endocytosis, effectively shielding it from nuclease degradation and minimizing immune system interference.^115^ He et al. used folate receptor targeting liposomes to deliver CRISPR plasmids that co-expressed Cas9 and sgRNA targeting the ovarian cancer-related DNA methyltransferase 1 (*DNMT1*) gene, which were safely injected into ovarian cancer cells.^116^ The expression of *DNMT1* gene was successfully down-regulated, thereby effectively restraining the tumor growth of paclitaxel-sensitive and drug-resistant ovarian cancer. Wang et al. designed multifunctional LNPs modified with pH-sensitive EGFR-targeting and nucleus-directed peptides for the efficient delivery of HuR CRISPR and epirubicin to human tongue squamous carcinoma SAS cells and SAS tumor-bearing mice.^96^ This approach enabled HuR gene knockout while significantly inhibiting tumor cell proliferation, metastasis, and drug resistance, which provided an innovative combination treatment option for conventional chemotherapy and gene editing systems. Overall, lipid-based nanoparticles offer a versatile and efficient platform for CRISPR-Cas9 delivery in oral cancer treatment.

Gold nanoparticles (AuNPs) represent a class of inorganic nanomaterials characterized by highly controllable size and uniform distribution compared with LNPs.^117^ The combination of Cas9, sgRNA, and AuNPs in a complex, followed by their entry into cells via a cholesterol-mediated membrane fusion process, which showed a considerable improvement in delivery efficiency and successful gene editing.^97^ CRISPR–Gold could deliver both Cas9 and Cpf1 ribonucleoproteins, and could edit the major cell types in the brain, including neurons, astrocytes and microglia.^98^ Moreover, the CRISPR-Gold system, a precisely engineered platform designed to target the metabotropic glutamate receptor 5 (mGluR5) gene, demonstrated remarkable efficacy in reducing local mGluR5 levels in the striatum following intracranial administration. This targeted gene modulation not only ameliorated neurobehavioral deficits associated with fragile X syndrome in mice but also established a valuable framework for advancing intracerebral gene therapy applications. These successful applications highlight the highly valuable nature of CRISPR-Gold, making it a promising candidate for targeted gene therapy in oral diseases.

Nanopolymers are generally prepared from natural or synthetic polymer materials. Polymeric nanoparticles have gained prominence in nucleic acid delivery due to their straightforward fabrication, structural stability, and favorable biosafety.^99^ The CRISPR-Cas9 system, when complexed with fluoridated polymer (PF33), formed a nanoscale core that could be encapsulated within a multifunctional shell composed of RGD-R8-PEG-HA (RRPH). This engineered RRPHC artificial virus demonstrated remarkable capabilities in efficient CRISPR-Cas9 delivery, enhanced lysosomal escape, and successful gene editing without additional nuclear localization signals.^100^ In summary, nanopolymers demonstrate great potential as stable and biocompatible carriers for gene editor delivery in oral disease treatments.

### 4.4 Other delivery ways

Hydrogels are three-dimensional polymeric networks composed of hydrophilic polymer chains that are interconnected and embedded within a water-rich environment ^118^. They have been applied in different fields, such as drug delivery, tissue regeneration, and biosensors. While various hydrogels have been extensively applied in oral tissue engineering, their integration with gene editing technologies has recently expanding the therapeutic scope in this field. However, a critical challenge for hydrogels in the oral cavity is the profound impact of masticatory forces. Continuous mechanical loading, including occlusal compression and lateral shear stress during chewing, can physically disrupt the hydrogel's cross-linked network. This structural deformation not only accelerates the bulk degradation of the hydrogel but also alters its swelling ratio, leading to uncontrolled, burst release of the encapsulated CRISPR-Cas cargo rather than the desired sustained local delivery.^119^ Therefore, engineering tough, mechanically resilient, or tissue-adhesive hydrogels is essential to withstand oral occlusal forces. To prevent mechanical-stress-induced matrix break and burst cargo leakage, advanced polymeric strategies have been actively developed. For instance, fabricating double-network hydrogels or interpenetrating polymer networks (IPNs) enables efficient masticatory energy dissipation, maintaining structural integrity under dynamic compression.^120^ Additionally, incorporating mussel-inspired catechol groups (such as polydopamine) or dynamic covalent linkages (such as schiff-base crosslinks) endows scaffolds with robust wet-tissue adhesion and rapid self-healing capabilities, ensuring sustained and localized gene-editor release within the active oral cavity.^121^, ^122^ Utilizing CRISPR-Cas9-mediated gene editing technology, researchers developed *Ndrg2*-knockout dendritic cells and incorporated them into pullulan-collagen hydrogel carriers. This advanced therapeutic platform enabled precise and sustained delivery of engineered cells to wound sites, significantly enhancing full-thickness wound repair in both non-diabetic and diabetic mouse models.^101^ The injectable hydrogel microsphere system, comprising methacrylic anhydride-modified hyaluronic acid combined with *in vivo FGF18* gene editing could promote cartilage regeneration, reduce inflammation, and inhibit ECM degradation both *in vitro* and *in vivo*, thereby representing a promising translational strategy for cartilage repair.^123^

Nano/micro fiber membranes were fabricated from natural polymers and synthetic polymers by multiple material manufacturing technology, including 3D bioprinting, microfluidics spinning, electrospinning and others.^124^ Due to superior performance, such as heightened mechanical strength, augmented surface-area-to-volume ratio, and distinctive functionalities^125^, nano/micro fiber membranes have been widely applied in biomedical fields, including dentistry. Building upon this, incorporating gene-editing agents into fibrous scaffolds can endow them with targeted therapeutic capabilities. The Cas9:sgRNA lipofectamine complexes were effectively immobilized onto PCL fiber scaffolds through polyDOPA-melanin-mediated adsorption, enabling localized and sustained non-viral delivery of Cas9:sgRNA complexes for useful applications in tissue regeneration.^102^ Zhang et al. developed multilayer nanofiber as delivery methods for CRISPR-Cas9 systems.^126^ An amphiphilic negatively charged self-assembling peptide (SAP) was coated onto PCL nanofibers was successfully functionalized onto PCL nanofibers through hydrophobic interactions, followed by the sequential assembly of pDNA complexes and cationic SAP+-RGD via electrostatic interactions. The system could achieve efficient loading and sustained release of Cas9/sgRNA plasmids to secrete GDNF for neurite outgrowth. The integration of gene editing techniques with nano/micro fiber membranes may provide important references for the treatment of oral mucosal diseases, periodontal membrane diseases, and others.

### 4.5 Potentially transferable delivery technologies for CRISPR systems

Though primarily designed for conventional drug delivery and tissue engineering in dentistry, highly optimized platforms such as nanoparticles, hydrogels, and fibrous membranes can be readily adapted for CRISPR-Cas delivery.

Lipid, inorganic, and polymeric nanoparticles are among the most extensively applied nanocarriers in oral disease management. For OSCC, liposomes enabled localized Erlotinib delivery for theranostic applications,^127^, ^128^ while gold nanoparticles (AuNPs) facilitate screening and radio-photothermal therapies.^129^ Polymeric variants also demonstrate robust translational capacity. Chitosan and PMPC-PDPA nanoparticles effectively deliver periodontitis antibiotics,^130^, ^131^ and PEG-PLA-PEG nanoparticles allow intralesional delivery of chemotherapeutics and Cetuximab for OSCC.^132^

Macroscopic biomaterials, including hydrogels and fibrous scaffolds, offer additional translatable strategies. In OSCC models, silver nanoparticle-incorporated adhesive hydrogels synergized with PD-1 inhibitors to augment anti-tumor immunity.^133^ Furthermore, photo-crosslinking hydrogels significantly accelerated mucosal wound healing via rapid tissue anchoring.^122^ In periodontal tissue engineering, electrospun fibrous membranes effectively regulated the proliferation and migration of gingival fibroblasts and promote osteogenesis.^134^, ^135^ These validated carriers could enable safe, targeted, and sustained release of gene editors within the oral microenvironment.

Achieving precise *in vivo* delivery of gene editors remains a paramount challenge. Optimizing these delivery tools are essential to bridge the gap between preclinical research and clinical application. Ultimately, developing effective delivery system will be pivotal to fully harness the therapeutic potential of gene editing for oral diseases.

## 5 The application status of gene editing technology

### 5.1 Advantages of gene editing in oral disease treatments

In the treatment of oral diseases, CRISPR-based gene editing therapy demonstrates distinct advantages over conventional interventions, particularly in providing personalized and durable solutions for diseases that lack effective long-term solutions.^17^ (Fig. 6) Firstly, gene editing technology exhibits targeted specificity. By acting directly on target sequences or specific cell populations, it offers precise molecular specificity that has the potential to mitigate damage to surrounding normal tissues compared to traditional therapies. Secondly, it enables precise genetic modification with high gene editing efficiency. Rather than directed or ablative strategies, this approach directly intervenes in the molecular pathogenesis of disease, providing the potential for permanent gene correction at the site of pathology. Thirdly, gene editing establishes a powerful framework for individualized treatment. Depending on the clinical requirements, therapies can be tailored through *in vivo* gene editing (e.g., injecting gene editing system directly into the patient) or *ex vivo* gene editing (e.g., transplanting edited cells back to patients). This customizable approach is especially promising for managing complex conditions like caries, periodontal disease, and oral cancer. Previous studies have shown that CRISPR-Cas9-based *in vivo* genome-wide screening can direct early therapeutic interventions in head and neck squamous cell carcinoma (HNSCC), particularly in OSCC, with high specificity and efficiency.^80^, ^136^ Furthermore, the CRISPR system has shown promising prospects for advancement by leveraging precise, molecularly targeted therapies in conjunction with other strategies to control the progression of oral diseases.

**Fig. 6:**
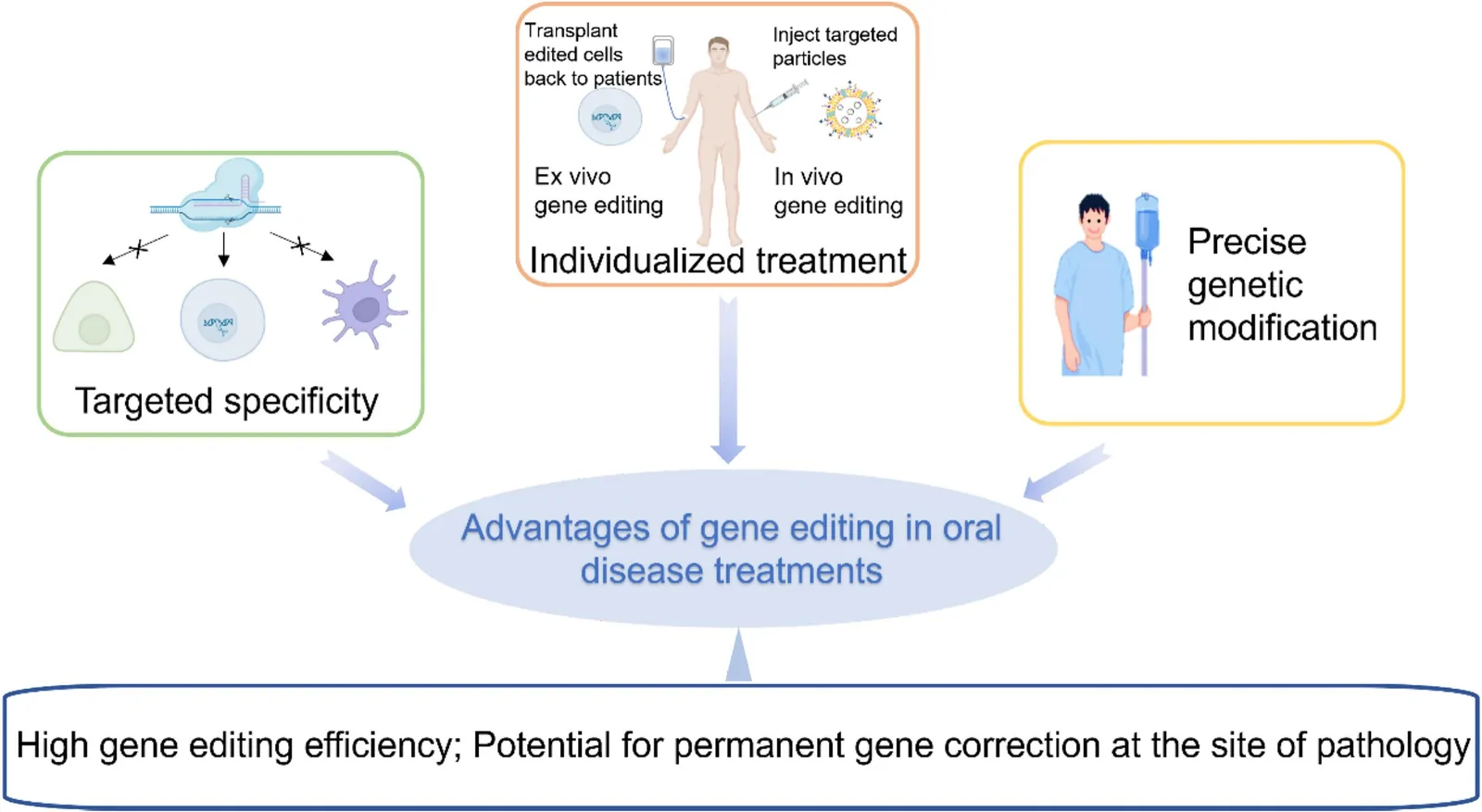
Advantages of gene editing in the field of oral disease treatments.

### 5.2 Challenges of gene editing

Despite its transformative potential, the inherent risk of off-target editing remains a critical safety hurdle for genomic therapies. Off-target sites span a wide spectrum, from those with only a single base pair mismatch to those featuring numerous consecutive mismatches or even insertions and deletions. Off-target cleavage at multiple genomic sites can induce genomic rearrangements, including deletions, inversions, and translocations, and activate DNA damage and stress response pathways. While off-target editing is widely recognized as a critical hurdle, a comprehensive safety evaluation must expand far beyond these general off-target effects. At the targeted loci, unintended consequences such as on-target large deletions, complex genomic rearrangements, and chromosomal translocations must be rigorously evaluated.^53^ Furthermore, *in vivo* therapeutic interventions carry the risks of cellular mosaicism, and prolonged nuclease expression can exacerbate both off-target activity and cellular toxicity. The introduction of exogenous components often elicits robust immune responses against Cas proteins or delivery vectors, thereby posing a dual threat to therapeutic efficacy and patient safety.^81^ Additionally, additional safety concerns including insertional mutagenesis, the inherent irreversibility of permanent DNA modifications, and unintended germline exposure demand rigorous oversight. Furthermore, oral cells exhibit distinct genomic instability compared to other tissues. Due to continuous exposure to environmental mutagens (e.g., tobacco, alcohol, and human papillomavirus) and a highly proliferative basal layer, oral mucosal cells often possess unique pre-existing genomic instability.^63^ This dynamic drastically alters DNA repair pathway choices, inherently favoring hyperactive NHEJ over HDR when compared to quiescent tissues like the liver.^137^ Consequently, following CRISPR-induced cleavage, this sensitized oral background results in an off-target signature that is significantly more prone to large structural variations, translocations, and complex indels than the predictable profiles observed in other somatic compartments.^53^ Specifically within the oral environment, gene editing of the dysbiotic microbiome raises the unique ecological risk of gene transfer among resident oral microorganisms.^29^

Improving the targeting accuracy of CRISPR-Cas9 system is a key issue for further development. Some related studies have made great progress. By constructing cell targeted delivery systems^138^, cell-specific promoter plasmids, tissue environment responsive release systems^82^, etc., the targeting of gene editing can be effectively improved. Yi et al. developed a fusogenic liposome featuring a galactose-modified membrane-fusogenic lipid coating, which could target liver sites by fusing with HCC tumor cells. The lipid nanoparticles transported interior CDK5- and PLK1-targeting CRISPR-Cas9 plasmid into the HCC cells effectively, resulting in significantly enhanced therapeutic efficacy against hepatocellular carcinoma.^138^ Zhao et al. designed a functional nano system containing the HSP70-promoter-driven CRISPR-Cas9, which activated the HSP70 promoter specifically under a 660 nm laser and enabled the specific expression of Cas9 protein for precise *PD-L1* gene disruption. This system could achieve precise spatiotemporal control of gene editing to reduce severe immune-related adverse events in the clinic.^83^ Zheng et al. designed biodegradable LNPs for the targeted delivery of proenzymes and therapeutic biomacromolecules, which demonstrated remarkable efficacy by controlling the degradation of LNPs in response to the tumor microenvironment.^82^ These highly controllable, environment-responsive nanocarriers provide a translatable framework for improving the targeting accuracy of CRISPR-Cas9 delivery systems. In brief, a variety of precise targeting strategies need to be continuously developed to improve the accuracy of gene editing.

Current gene editing interventions are largely constrained by invasive procedures, such as tissue biopsies and localized administration, or rely on complex *ex vivo* cell manipulations and viral delivery systems. Furthermore, the long-term safety and cost-effectiveness of these treatments vary considerably depending on the technological platforms employed.^22^ Therefore, substantial efforts are still required to facilitate the routine clinical translation of gene editing technologies.

### 5.3 Ethical implications

As gene editing technologies advance toward clinical translation, somatic editing must be clearly distinguished from heritable germline editing, with each governed by its own ethical and regulatory frameworks.^139^ Currently, the application of gene editing in oral diseases, such as targeting OSCC, periodontal tissues, or maxillofacial bone defects, is strictly confined to somatic editing. In these clinical contexts, gene editing and its associated risks remain highly localized and controllable within the individual patient. However, an absolute ethical consensus requires that these interventions must be rigorously regulated to strictly prohibit any inadvertent germline exposure. Robust legislative and regulatory guidelines are required to ensure that gene editing in oral healthcare does not induce heritable alterations to the human genome, thereby maintaining the highest standards of biosafety and clinical ethics.^8^, ^140^

## 6 Conclusion

In conclusion, gene editing technology is closely associated with the occurrence, development, and prognosis of oral diseases. It is significant to explore important pathogenesis and valuable molecular targets for the treatment and prognosis of oral diseases. Gene editing technology combined with other approaches can solve the problems existing in traditional treatment methods. All in all, the development of gene editing technology brings new hope for the treatment of oral diseases.

Gene editing technology is a promising therapeutic approach, which can bring novel ideas for the treatment of oral diseases. The current research on gene editing technology in the field of dentistry is still in the preliminary stage, but its development prospects are broad. Overall, it must be emphasized that the current evidence for gene editing in oral diseases remains predominantly preclinical, and direct human clinical evidence is still extremely limited. In addition, gene editing technology can offer various therapeutic strategies for oral diseases, potentially advancing the field of dentistry. Therefore, more extensive and rigorous investigations are still required moving forward.

With the development of research and application, the potential of gene editing will continue to be explored. At the same time, we need to pay more attention to its safety, stability and ethical issues. If the gene editing technology is improperly applied, it may cause damage to the human genome and fundamentally interfere with the stability of the human species. Gene editing technology has the potential to drive remarkable progress and bring substantial benefits to society, provided that its development and application are carefully guided by ethical considerations and legal regulations.

To achieve the effective application of gene editing for oral disease treatments in the future, various cutting-edge strategies and interdisciplinary approaches must be prioritized. First, the integration of artificial intelligence (AI) requires deeper development; beyond basic predictions, machine learning models will be instrumental in accurately mapping off-target effects, optimizing sgRNA designs, and establishing personalized delivery pathways based on patient-specific genetic profiles. Second, through technological innovations, such as exploring new editing systems and designing novel delivery carriers, the development of gene editing technology can be effectively promoted. Additionally, multidisciplinary collaboration is essential to accelerate clinical translation, encompassing the conduct of clinical trials and the establishment of standardized evaluation standards. Furthermore, the successful transition of these technologies to the clinic hinges upon innovations in *in vivo* delivery systems. The development of stimuli-responsive nanoparticles, targeted viral vectors, and tissue-adhesive hydrogels is crucial for ensuring the localized, sustained, and biocompatible release of gene editing machineries within the highly complex oral microenvironment. Finally, the optimization of combinatorial therapeutic strategies warrants thorough investigation. Integrating CRISPR-based interventions with established clinical modalities—such as PD-1 immune checkpoint blockade, conventional chemotherapy, or photothermal therapy—offers a robust paradigm to circumvent drug resistance, augment immune responses, and achieve synergistic efficacy against oral squamous cell carcinoma (OSCC). All in all, continuous research and innovation play indispensable roles in realizing the widespread application of gene editing technology in the field of oral health.

## CRediT authorship contribution statement

**Yihan Wang:** Writing – original draft, Investigation. **Xuesong Li:** Writing – original draft, Investigation. **Chunpeng Liu:** Writing – review & editing, Methodology. **Bing He:** Methodology, Investigation. **Yiming Liu:** Methodology, Investigation. **Qingyan Liu:** Methodology, Investigation. **Chenyu Liu:** Methodology, Investigation. **Daiyun Chen:** Supervision, Funding acquisition. **Shufang Wang:** Writing – review & editing. **Lin Zhang:** Writing – review & editing, Validation, Supervision, Methodology, Funding acquisition.

## Declaration of competing interest

The authors declare that they have no known competing financial interests or personal relationships that could have appeared to influence the work reported in this paper.
